# Insights into Metabolic Reactions of Semi-Dwarf, Barley Brassinosteroid Mutants to Drought

**DOI:** 10.3390/ijms21145096

**Published:** 2020-07-19

**Authors:** Damian Gruszka, Ewa Pociecha, Barbara Jurczyk, Michał Dziurka, Jakub Oliwa, Iwona Sadura, Anna Janeczko

**Affiliations:** 1 Institute of Biology, Biotechnology and Environmental Protection, Faculty of Natural Sciences, University of Silesia, Jagiellonska 28, 40-032 Katowice, Poland; 2 Department of Plant Breeding, Physiology and Seed Science, University of Agriculture in Krakow, 30-239 Krakow, Poland; rrchilmo@cyf-kr.edu.pl (E.P.); barbara.jurczyk@urk.edu.pl (B.J.); 3 The Franciszek Gorski Institute of Plant Physiology, Polish Academy of Sciences, 30-239 Krakow, Poland; m.dziurka@ifr-pan.edu.pl (M.D.); saduraiwona@gmail.com (I.S.); a.janeczko@ifr-pan.edu.pl (A.J.); 4 Department of Chemistry and Biochemistry, Institute of Basic Sciences, University of Physical Education, 31-571 Krakow, Poland; jakuboliwa@gmail.com

**Keywords:** barley, brassinosteroid, drought, enzymatic antioxidants, semi-dwarf, soluble sugars

## Abstract

The roles of endogenous brassinosteroids (BRs) in the modulation of reaction to drought and genetic regulation of this process are still obscure. In this study, a multidirectional analysis was performed on semi-dwarf barley (*Hordeum vulgare*) Near-Isogenic Lines (NILs) and the reference cultivar “Bowman” to get insights into various aspects of metabolic reaction to drought. The NILs are defective in BR biosynthesis or signaling and displayed an enhanced tolerance to drought. The BR metabolism perturbations affected the glucose and fructose accumulation under the control and stress conditions. The BR metabolism abnormalities negatively affected the sucrose accumulation as well. However, during drought, the BR-deficient NILs accumulated higher contents of sucrose than the “Bowman” cultivar. Under the control conditions, accumulation of transcripts encoding antioxidant enzymes ascorbate peroxidase (HvAPX) and superoxide dismutase (HvSOD) was BR-dependent. However, during drought, the accumulation of *HvAPX* transcript was BR-dependent, whereas accumulations of transcripts encoding catalase (HvCAT) and HvSOD were not affected by the BR metabolism perturbations. The obtained results reveal a significant role of BRs in regulation of the HvAPX and HvCAT enzymatic activities under control conditions and the HvAPX and HvSOD activities during physiological reactions to drought.

## 1. Introduction

Crop plants are frequently exposed to various environmental stresses. Drought poses a major factor limiting plant growth, development and reproduction, thus negatively affects yield [1,2,3,4,5,6]. It was reported in several monocot and dicot crop species that drought may result in a drastic decrease in the crop yield by more than 50% [7,8]. Moreover, recent predictions indicate that in the near future crop cultivation in several regions of the world will encounter even more severe environmental conditions due to global climate change. Consequently, the yield losses caused by drought will become even more significant [9,10,11]. In the past, high yielding varieties were developed for optimal environmental conditions. However, given the current climate changes, priority should be given to developing and breeding the stress tolerant cultivars [4,8].

In the course of evolution, plants have developed various adaptive mechanisms that allow an efficient reaction to drought [8]. One of the most important mechanisms underlying plant adaptation to drought is mediated by alteration in concentrations of phytohormones, which play a key role in modulation of development and physiology [10]. Brassinosteroids (BRs) constitute a group of phytohormones which are known to regulate a broad range of developmental and physiological processes, both directly or through a convergence of BR signaling with response pathways of other phytohormones and stress cues [12]. These interactions are crucial for a maintenance of balance between the processes of plant growth and stress tolerance [13,14,15]. In previous experiments, the role of BRs in regulation of plant reaction to drought was analyzed mostly through application of exogenous BRs or BR-biosynthesis inhibitors [16,17,18,19,20,21]. Although such experiments are useful for development of BR-based agrochemicals for plant protection against stressors, the knowledge about the role of endogenous BRs in the modulation of plant reaction to drought and the genetic regulation of this process remains limited [22,23,24,25]. It should be kept in mind that the plant reaction to drought varies between species and depends on plant growth stage and environmental factors [26]. This makes an interpretation of the exogenous BR treatment-based results even more difficult. Importantly, the effect of the BR treatment on stress tolerance depends on the exogenous BR concentration, and excessive BR concentrations may have detrimental consequences as optimal progress of the BR signaling relies on appropriate BR concentration [27]. Moreover, during these studies, the exogenous BRs were applied at relatively high concentrations, which are known to induce various physiological responses [28]. Hence, secondary effects could be triggered based on the crosstalk between the BR signaling pathway and signalosomes of other phytohormones and environmental stimuli [15].

Importantly, recent reports in *Arabidopsis thaliana* (thale cress) and monocot species *Brachypodium distachyon* (purple false brome), *Hordeum vulgare* (barley) and *Oryza sativa* (rice) brought intriguing results indicating that mutants defective in the BR metabolism show an enhanced tolerance to drought [28,29,30,31,32]. Moreover, it was reported that an enhancement of the BR signaling negatively regulates the drought resistance [33]. However, drought tolerance is a complex phenomenon regulated by a number of genes [11,34]. It is also known that annual crop plants adapt to drought by decreasing their leaf size and delaying flowering [35]. Generally, drought suppresses growth of crop plants [36,37], and an increased root-to-shoot ratio is one of the stress adaptations [4,38]. Therefore, semi-dwarf mutants of cereal crops may be regarded as pre-adapted to the stress through the drought-avoidance strategy [39,40]. Indeed, our previous study indicated that the semi-dwarf barley mutants defective in the BR metabolism may be considered as an alternative in the future breeding programs [41]. The semi-dwarf barley BR mutants characterized by reduced biomass of the above-ground part but normal architecture of root system [41] exhibited delayed wilting under the drought conditions when compared with the reference cultivar. The mutants were also less affected by the drought-related growth reduction than plants of parent cultivars [31,32]. Moreover, we applied the semi-dwarf barley BR mutants as a tool to obtain insights into the phytohormonal homeostasis and physiological reaction during the drought stress. A significant increase in accumulation of biologically active BR, castasterone, was reported in reaction to drought. This indicated that castasterone may be regarded as a stress-induced compound. It was also reported that concentration of one of the bioactive forms of gibberellins, GA_7_, is BR-dependent and significantly drought-induced in barley. Similarly, it was shown that the regulation of jasmonic acid homeostasis in barley is also a BR-dependent process [31]. More recently, analysis of the reaction of the semi-dwarf barley BR mutants to drought indicated that the endogenous BRs play an important role in regulation of non-enzymatic antioxidants—ascorbate, glutathione and tocopherols [42]. Considering that the endogenous BRs regulate the homeostasis of non-enzymatic antioxidants, we hypothesized that the disturbances in BR metabolism may also influence antioxidant enzymes, and this hypothesis was verified in the presented study. It is known that exposure to drought initially results in the oxidative damage caused by formation of reactive oxygen species (ROS), which are the major cause of decline in crop productivity [8,39,43,44]. However, the cellular concentration of ROS is precisely controlled by the antioxidant system to maintain redox homeostasis, as at low concentration ROS play a role as stress signaling components [43,45,46,47]. Determining the influence of endogenous BRs and BR signaling on function of the antioxidant enzymes is particularly important, as it was reported that the enzymatic defense is more effective than the non-enzymatic one [6,48]. The major enzymes involved in this antioxidant response include catalase (CAT), superoxide dismutase (SOD) and ascorbate peroxidase (APX). The enzymes may directly scavenge ROS or function indirectly by regulating the non-enzymatic defense [20,49]. Moreover, ROS constitute a point of integration of the BR signaling with other hormonal, developmental and stress signaling pathways [25,50]. Thus, research on the hormone-dependent regulation of the ROS metabolism and activity of the enzymes maintaining the cellular redox balance is required, especially because understanding of these aspects is still scarce, also in barley, which is an important crop species [4,51]. Therefore, in the present study, we performed analysis of the enzymatic antioxidants at the transcriptional and protein activity level in the semi-dwarf, barley BR mutants exposed to drought. 

One of the key physiological processes which are negatively affected by drought is photosynthesis [18,52]. It is known that BRs upregulate the RuBisCO activity and enhance the net photosynthesis rate [19,32,53,54,55]. Under abiotic stresses, BRs also regulate the photosynthetic light reactions through maintaining energy flux efficiency within photosystem II and preventing loss of photosynthetic pigments [56,57]. However, the exact mechanism of the BR-dependent regulation of photosynthetic capacity is not fully elucidated [55]. Therefore, in this study, we analyzed the RuBisCO activity in the semi-dwarf BR mutants under the control conditions and changes in the RuBisCO activity which were caused by drought. Determining the profile of RuBisCO activity under drought is particularly important, considering the potential application of the semi-dwarf barley BR mutants in the future breeding programs during the approaching global climate changes. It is also known that drought brings about a decrease in the sucrose content, which reduces the source-to-sink export rate [58,59]. However, it has also been reported that soluble sugars (glucose and sucrose) may play a dual role in terms of the ROS homeostasis through enhancing the ROS production or participating indirectly in the ROS scavenging. Moreover, the sugars are also involved in the regulation of genes encoding the antioxidant enzymes, such as SOD [60,61,62]. Thus, sugars play a pivotal role in maintaining the osmotic potential [62,63] and in the regulation of redox homeostasis [60,64]. Moreover, a crosstalk between the glucose and the phytohormone signaling pathways regulates various physiological processes through balancing the carbon availability and plant development [65]. Interestingly, glucose alters expression of the majority (72%) of the BR-regulated genes, including key components of the BR signaling pathway [66,67,68]. It was also reported that BRs are indispensable for the sugar-induced hypocotyl elongation [69]. However, further research is still needed to fully uncover the intricacies of the glucose–BR crosstalk which regulates plant growth and productivity [68]. In fact, studies revealing specific metabolic reactions to drought remain limited, even in the model plant species [70]. Taking into account the ongoing global climate change, elucidating this interconnection under the drought conditions is particularly important. Therefore, in the study presented here, we performed a multidirectional analysis aimed at shedding light on the key metabolic mechanisms of the drought response. Owing to application of the semi-dwarf barley BR mutants in the study, the role of endogenous BRs in regulation of these processes was elucidated.

## 2. Results

### 2.1. Dynamics of Plant Growth under the Control and Drought Conditions

**Figure 1:**
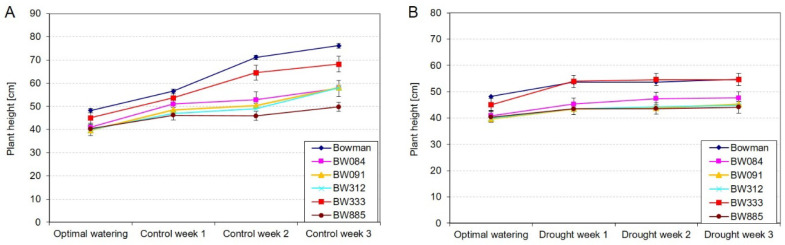
Dynamics of plant growth under the conditions of optimal watering (control) (**A**) and during exposure to the three-week drought period (**B**); *n* = 6 plants per genotype at each time point. The mean values are presented for each genotype, with error bars representing standard deviation. Details are given in the text.

To characterize the dynamics of growth of the analyzed genotypes under the control and drought conditions and to get insights into the mechanism underlying the delayed wilting which is exhibited in reaction to drought by the semi-dwarf NILs when compared with the “Bowman” cultivar [31], we measured a rate of plant growth under the optimal watering conditions (the fourth week of plant vegetation) and during the next three weeks of either further vegetation under the optimal watering conditions (control) or three weeks of increasing water deficit. During vegetation under the optimal watering conditions, throughout the whole experiment, plants of the semi-dwarf NILs at each of the analyzed time points were significantly (*p* ≤ 0.05) shorter than plants of the “Bowman” cultivar. Moreover, the rate of growth of the semi-dwarf NILs between Time Point 1 (optimal watering) and Time Point 4 (Control Week 3) was significantly (*p* ≤ 0.05) lower than that of the “Bowman” plants (Figure 1A). From Time Point 1 to Time Point 4, plants of the “Bowman” cultivar increased their height by more than 58% (at Time Point 4, their height reached more than 158% of the value reported for this genotype at Time Point 1). As far as the semi-dwarf NILs are concerned, the increase in their height reported between Time Point 1 and Time Point 4 reached from 25% (in BW885) to 51% (in BW333) and was significantly (*p* ≤ 0.05) lower than the respective increase reported in the “Bowman” cultivar. At Time Point 4, the height of the “Bowman” plants was from 111% to 152% of height of the BW333 and BW885 plants, respectively (Figure 1A).

The water deficit significantly altered the dynamics of plant growth, particularly the rate of growth of the “Bowman” plants. Between Time Point 1 (optimal watering) and Time Point 4 (Drought Week 3) plants of the “Bowman” cultivar increased their height by 14%. In the NILs, the increase in plant height reported between Time Point 1 and Time Point 4 reached from 10% (in BW885) to 22% (in BW333) (Figure 1B). More importantly, at Time Point 4, the height of the “Bowman” plants was from 100% of height of the BW333 plants (i.e., plants of the “Bowman” cultivar and the BW333 line were of the same height at this time point) to 125% of height of the BW885 plants (Figure 1B). From Drought Week 1 to Drought Week 3, the differences reported between the “Bowman” cultivar and the BW333 line were not statistically significant. Therefore, taking into account both the rate of plant growth and comparison of plant height among the genotypes at the end of drought stress period (Time Point 4), it may be postulated that plants of the “Bowman” cultivar were more negatively affected by the stress than plants of the semi-dwarf NILs.

### 2.2. Different Profiles of Drought-Induced Alteration in the Relative Water Content

**Figure 2:**
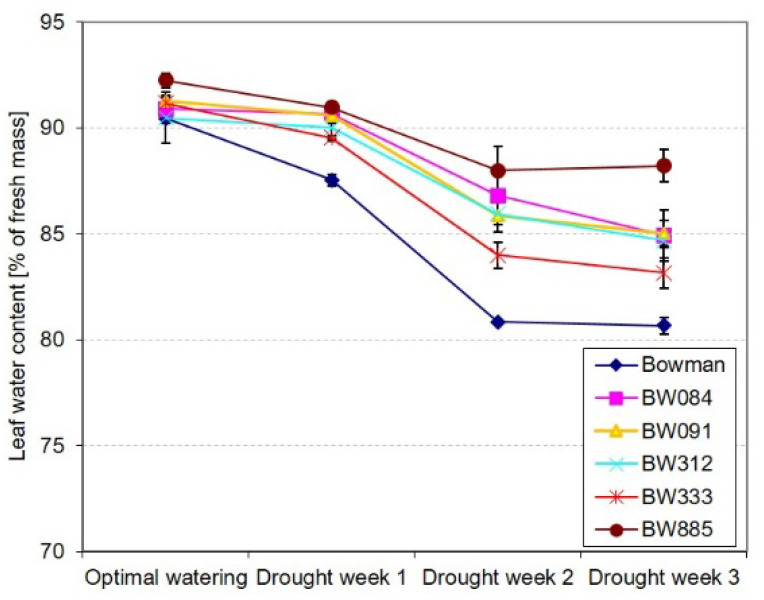
Changes in the leaf water content evoked by exposure of plants to the three-week drought period; *n* = 12 plants per genotype at each time point. The mean values are presented for each genotype, with error bars representing standard deviation. Details are given in the text.

To further verify whether plants of the “Bowman” cultivar were more negatively affected by the stress than plants of the semi-dwarf NILs, an analysis of changes in the relative water content in leaves of the genotypes was performed during the three-week period of increasing water deficit. Under the optimal watering conditions (Time Point 1), the relative water contents in leaves were similar in all analyzed genotypes (differences were not significant). As expected, the increasing water deficit resulted in a decrease of the relative water contents in leaves of the genotypes. However, the decline in the relative water content was the steepest in the “Bowman” cultivar, particularly between Time Point 1 (optimal watering) and Time Point 3 (Drought Week 2). In the “Bowman” cultivar between Time Point 1 (optimal watering) and Time Point 4 (Drought Week 3), the relative water content was decreased by 10%, whereas, in the NILs, the decrease ranged between 4% (in BW885) and 8% (in BW333). At Time Point 4, in the analyzed semi-dwarf NILs, the relative water contents were from 102% (BW333) to 108% (BW885) of the value reported in the “Bowman” cultivar (Figure 2). Further, according to our visual observations, in the “Bowman” plants, the wilting symptoms were the earliest visible (at the beginning of Drought Week 2). Upon the drought period, the wilting was observed in the first three leaves of the “Bowman” plants, and the first and second leaves were completely dried. At the same time, a weaker effect was reported in the BW333 plants (only the first leaf was dried). In the other BR mutants, the first leaves showed only partial wilting. A clear difference between the “Bowman” plants and the semi-dwarf BR mutants was reported in the third leaves: after the three weeks of drought, wilting of the third leaves was observed only in the “Bowman” plants. The leaf injuries of the “Bowman” and BR mutant plants at the end of the experiment, after the three weeks of drought, are shown in the Supplementary Materials (Figure S1).

### 2.3. Leaf Reflectance Parameters in Optimally Watered and Drought Stressed Plants 

**Figure 3:**
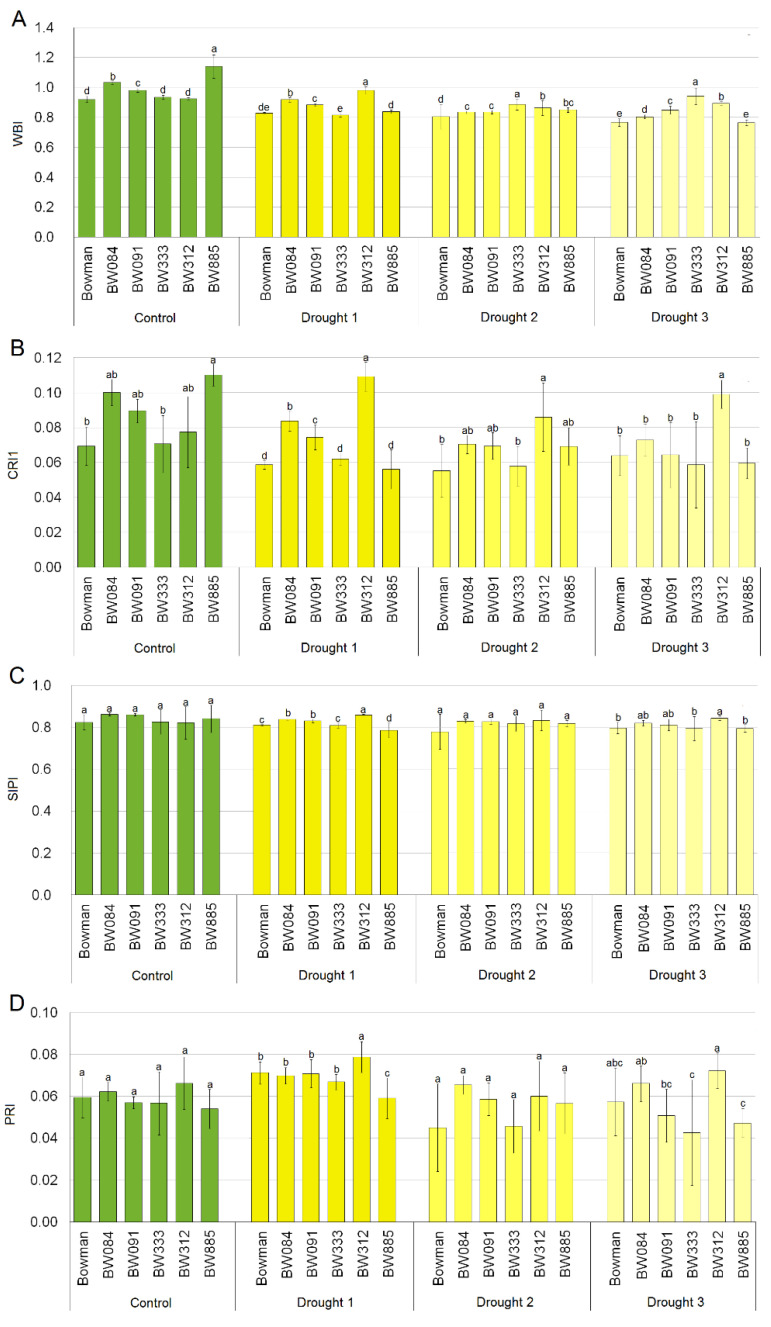
The values of the following parameters in the analyzed genotypes under the optimal watering conditions (control) and during the three-week drought period: WBI (**A**); CRI1 (**B**); SIPI (**C**); and PRI (**D**). *n* = 7 replicates per genotype at each time point. The mean values are presented for each genotype, with error bars representing standard deviation. The mean values (±SD) marked with the same letters (separately for the control and drought-stressed plants) are not significantly different, according to the Duncan’s test (*p* ≤ 0.05). Details are given in the text.

In control plants, leaf hydration estimated by the WBI values was different between the analyzed genotypes (Figure 3A). The lowest WBI values were found in plants of the reference cultivar (Bowman) as well as in the BW312 and BW333 mutants, and the highest in BW885. After the first and second weeks of drought, a decrease in the WBI values was observed (except for the BW333 and BW312 lines). After three weeks of drought, the WBI values of the analyzed NILs were significantly higher than in the “Bowman” cultivar, on average by 14%.

The total carotenoid content was determined based on the average CRI1 values (Figure 3B). In the leaves of plants growing under the control conditions, the content of carotenoid pigments in plants of the BW084, BW091 and BW885 lines was higher than in the “Bowman” cultivar. In the following weeks of drought, the differences between genotypes were decreasing, but not in the BW312 line. After three weeks of drought, in plants of the BW312 line, a significantly higher CRI1 value was observed when compared with the other genotypes (by 55% when compared with the “Bowman” cultivar). The ratio of carotenoids to chlorophyll (SIPI, Figure 3C) remained similar in all analyzed genotypes under both the control and stress conditions.

The quantum yield of photosynthesis associated with changes in the composition of xanthophyll pigments (PRI) under the control conditions did not differ significantly between the “Bowman” cultivar and the NILs (Figure 3D). After the first and third week of drought only the BW312 mutant showed a significantly higher PRI value in comparison with the “Bowman” cultivar (by 11% and 26%, respectively). In the other NILs, the PRI values during drought did not differ significantly from the “Bowman” value. Reflectance signatures of the analyzed genotypes under the control conditions and during the three-week drought period are shown in Supplementary Materials (Figure S2).

### 2.4. The RuBisCO Activity and Its Drought-Induced Alteration 

**Figure 4:**
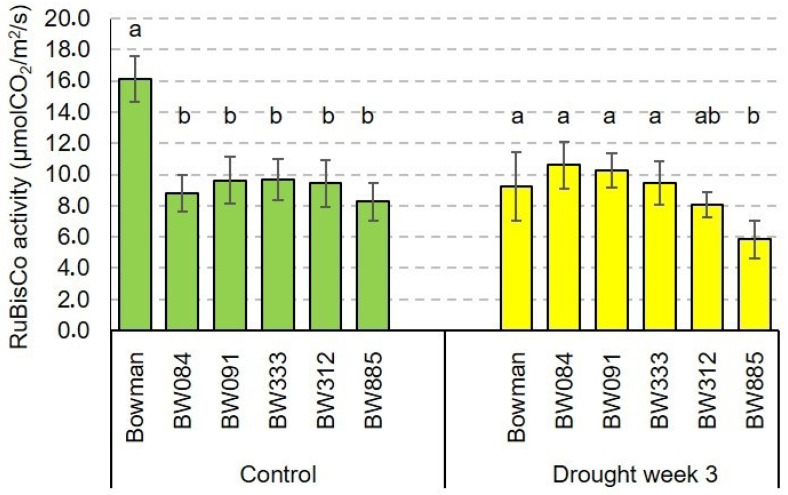
The RuBisCO activity in the analyzed genotypes under the control and drought conditions; *n* = 3 replicates per genotype per growth condition. The mean values are presented for each genotype, with error bars representing standard deviation. The mean values (±SD) marked with the same letters (separately for the control and drought-stressed plants) are not significantly different, according to the Duncan’s test (*p* ≤ 0.05). Details are given in the text.

It is known that carbon assimilation is a fundamental process both during growth under optimal conditions, but also during reaction of plant metabolism to stress conditions. Therefore, it was also important to determine the RuBisCO activity in the analyzed genotypes under the control and drought conditions. Under the control conditions, the RuBisCO activity was significantly higher in the “Bowman” cultivar when compared with the BR-deficient NILs. The BR-deficient NILs displayed very similar values of the RuBisCO activity. Under the control conditions, the RuBisCO activity in the “Bowman” cultivar was from 166% to 195% of the values reported in the BR-deficient NILs BW333 and BW885, respectively (Figure 4). 

Interestingly, drought resulted in a very significant decline in the RuBisCO activity specifically in the “Bowman” cultivar. In this genotype, the RuBisCO activity under the drought conditions was decreased by 43% when compared with the control value. Generally, the stress did not change the RuBisCO activity significantly in the analyzed NILs. Consequently, in all the analyzed genotypes (“Bowman” and the analyzed NILs), the values of the RuBisCO activity under drought stress were at a similar level (Figure 4).

### 2.5. Soluble Sugar Accumulation Profiles under the Control and Drought Conditions

**Figure 5:**
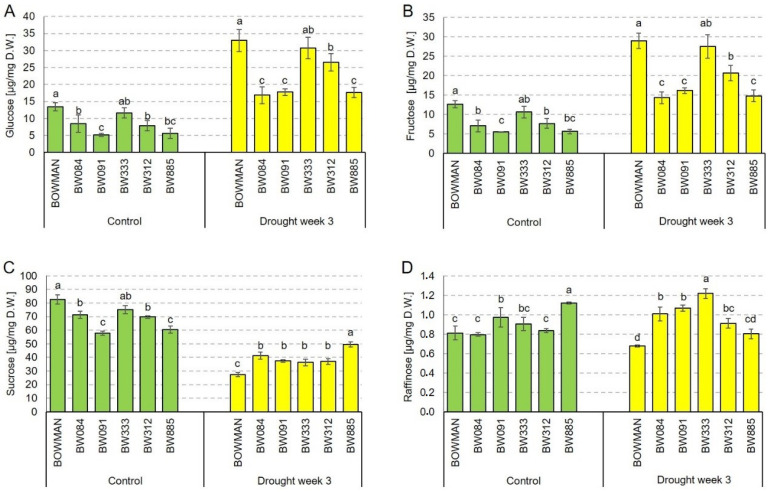
The accumulations in the analyzed genotypes under the control and drought conditions: of glucose (**A**); fructose (**B**); sucrose (**C**); and raffinose (**D**), *n* = 4 replicates per genotype per growth condition. The mean values are presented for each genotype, with error bars representing standard deviation. The mean values (±SD) marked with the same letters (separately for the control and drought-stressed plants) are not significantly different, according to the Duncan’s test (*p* ≤ 0.05). Details are given in the text.

Taking into account the above results of the RuBisCO activity, the next analysis was aimed to determine the soluble sugar accumulation profiles in the analyzed genotypes under the control and drought conditions. In this study, accumulation profiles of glucose, fructose, sucrose and raffinose were determined. As far as the glucose accumulation is concerned, under the control conditions, the highest content of this compound was reported in the “Bowman” cultivar. In the analyzed NILs, the accumulation of glucose was reduced to a various degree when compared with the “Bowman” cultivar. The glucose accumulation in the NILs was from 38.6% (in BW091) to 86.4% (in BW333) of the value reported in the “Bowman” cultivar (Figure 5A). The drought stress resulted in a significant (at least two-fold) increase in the accumulation of glucose in all analyzed genotypes. However, the highest content of glucose was reported in the “Bowman” cultivar. In the analyzed NILs, the accumulation of glucose was reduced to various degrees when compared with the “Bowman” cultivar. The glucose accumulation in the NILs was from 51% (in BW084) to 93% (in BW333) of the “Bowman” value. The results indicate that perturbations in the BR biosynthesis or signaling negatively affected the glucose accumulation under the control and drought conditions. It may also be concluded that drought stimulated the accumulation of glucose, and the BR-deficient mutants retained the ability to react to the stress accordingly (Figure 5A). 

Accumulation of fructose showed a very similar profile. Under the control conditions, the highest content of this compound was reported in the “Bowman” cultivar. In the analyzed NILs, the accumulation of fructose was reduced to a various degree when compared with the reference value. The accumulation of fructose was from 43% (in BW091) to 84% (in BW333) of the value reported in the “Bowman” cultivar (Figure 5B). The drought stress resulted in a significant (at least two-fold) increase in the accumulation of fructose in all the analyzed genotypes. Under the stress conditions, the highest content of fructose was reported in the “Bowman” cultivar. In the analyzed NILs, the accumulation of this compound was diminished by a various degree when compared with the “Bowman” value. Under the drought conditions, the fructose content was from 49% (in BW084) to 95% (in BW333) of the “Bowman” value. The results indicate that perturbations in the BR biosynthesis or signaling negatively affected the fructose accumulation under the control and drought conditions. Moreover, drought stimulated the accumulation of fructose and this physiological reaction to the stress was maintained in the analyzed NILs (Figure 5B). Generally, the results indicate that perturbations in the BR metabolism influence the profiles of glucose and fructose accumulation in a similar manner under both the control and stress conditions.

As far as the sucrose accumulation is concerned, under the control conditions, the highest content of this disaccharide was reported in the “Bowman” cultivar. In the analyzed NILs, the sucrose content was reduced by a various degree when compared with the reference value. The sucrose content was from 69% (in BW091) to 90% (in BW333) of the value reported in the “Bowman” cultivar (Figure 5C). Thus, under the control conditions, the genotype-dependent profile of sucrose accumulation was similar to the glucose and fructose accumulation profiles. However, in contrast to the profile of accumulation of glucose and fructose in reaction to drought, the stress conditions induced a significant reduction in the sucrose content in all the analyzed genotypes. Interestingly, the degree of the decrease in the sucrose accumulation was different among the analyzed genotypes. The most significant decline in the sucrose content (by 66%) was reported in the “Bowman” cultivar. However, in the analyzed NILs, the drought-induced reduction in the sucrose content was in the range 18–51%. Generally, the profile of sucrose content under the drought conditions was inversely correlated with the profile of accumulation of this compound under the control conditions. Interestingly, under the control conditions, the deficiency in the BR metabolism negatively affected the sucrose accumulation, whereas, under the drought conditions, the NILs accumulated higher contents of sucrose than the “Bowman” cultivar (Figure 5C).

As far as the raffinose accumulation is concerned, content of this trisaccharide was the lowest of all analyzed soluble carbohydrates. Under the control conditions, some differences were reported between the analyzed genotypes; however, no tendency in the variation was determined. Generally, the raffinose contents observed in the NILs were at the level reported in the “Bowman” cultivar or higher (Figure 5D). In contrast to the accumulation profiles of glucose, fructose and sucrose (see above), the drought stress did not lead to a significant change in the raffinose accumulation profiles in the analyzed genotypes. Some changes in the accumulation of raffinose in each of the analyzed genotypes were reported; however, no general tendency of the alterations could be determined (Figure 5D).

### 2.6. Accumulation Profiles of the HvAPX, HvCAT2 and HvSOD1 Transcripts Encoding Antioxidant Enzymes

**Figure 6:**
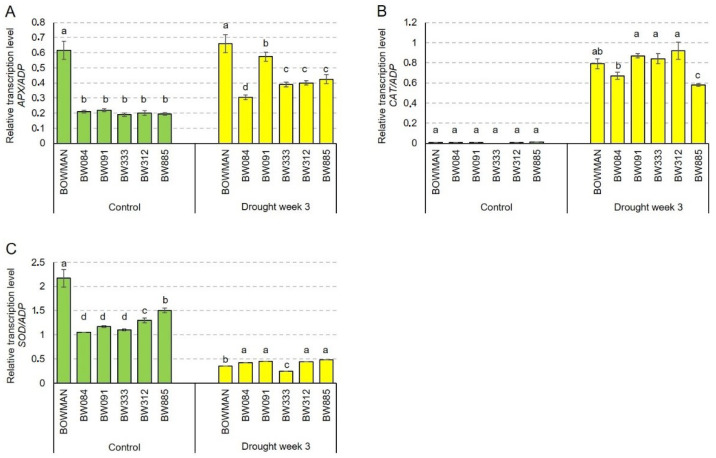
The relative transcription levels of the following genes in the analyzed genotypes under the control and drought conditions: *HvAPX* (**A**); *HvCAT* (**B**); and *HvSOD* (**C**). *n* = 3 replicates per genotype per growth condition. The mean values are presented for each genotype, with error bars representing standard deviation. The mean values (±SD) marked with the same letters (separately for the control and drought-stressed plants) are not significantly different, according to the Duncan’s test (*p* ≤ 0.05). Details are given in the text.

In our previous study, it was reported that endogenous BRs (and perturbations in their biosynthesis or signaling) affect accumulations of the non-enzymatic antioxidants under control and drought conditions [42]. Therefore, in the present study, we wanted to verify a hypothesis that abnormalities in the BR metabolism may influence the enzymatic antioxidants as well. To verify the hypothesis, the following parameters were analyzed in the current study: transcriptional level of the *HvAPX, HvCAT2*, and *HvSOD1* genes and enzymatic activity of the encoded proteins. As far as the *HvAPX* transcription level is concerned, under the control conditions, all the analyzed NILs showed very similar values of this parameter, and the transcription level was significantly (three times) lower than the respective value reported in the “Bowman” cultivar. These results indicate that abnormalities in the BR biosynthesis or signaling negatively affect transcription of the *HvAPX* gene under the control conditions (Figure 6A). The drought stress caused an increase in the *HvAPX* transcription level. However, a degree of the increase differed between the genotypes. In the “Bowman” cultivar, the drought stress induced the increase in the *HvAPX* transcription by 7%. However, in the analyzed NILs, the stress-induced increase in transcription ranged between 45% (in BW084) and 161% (in BW091) of the control values. It indicates that, in the analyzed NILs, the stress-induced increase in the *HvAPX* transcription was much more significant than in “Bowman”. Nevertheless, despite this differential increase in the *HvAPX* transcription under the drought conditions the values observed in the analyzed NILs were significantly lower than in the “Bowman” cultivar (Figure 6A). The results indicate that the abnormalities in BR biosynthesis and signaling negatively affect accumulation of the *HvAPX* transcript both under the control and drought conditions.

Interestingly, under the control conditions, transcription of the *HvCAT* gene was at a relatively very low level in all the analyzed genotypes (Figure 6B) when compared with the *HvAPX* transcription level (transcription levels of the genes analyzed in this study were compared with the same reference gene *HvADP*). No statistically significant difference in the *HvCAT* transcription between the analyzed genotypes was observed. However, transcription of the *HvCAT* gene was significantly increased by the drought stress in all the analyzed genotypes. Under the stress conditions, values reported in the majority of NILs were comparable with the reference value observed in the “Bowman” cultivar, and no tendency in the variation was detected (Figure 6B). This indicates that transcription of the *HvCAT* gene in barley was significantly stimulated by drought, but abnormalities in the BR biosynthesis or signaling did not seem to influence the profile of *HvCAT* transcript accumulation. 

As far as the *HvSOD* transcript accumulation is concerned, under the control conditions, the values reported in the NILs were significantly lower than in the “Bowman” cultivar. The values observed in the NILs was from 48% (in BW084) to 69% (in BW885) of the “Bowman” value (Figure 6C). Surprisingly, drought led to a significant decline in the *HvSOD* transcript accumulation in all analyzed genotypes. Moreover, under the stress conditions, the relative transcription level of the *HvSOD* gene was comparable in all analyzed genotypes, and no tendency in the variation was detected (Figure 6C). The results indicate that perturbations of the BR biosynthesis or signaling negatively affected the *HvSOD* transcript accumulation under the control conditions. However, the abnormalities in the BR metabolism did not influence on the *HvSOD* gene transcription under the drought conditions. Concluding, the *HvAPX, HvCAT2* and *HvSOD1* transcript accumulations showed diverse profiles. The accumulation of the *HvAPX* and *HvSOD* transcripts under the control conditions is dependent on the BR biosynthesis and signaling. Drought induced the (moderate) increase in the *HvAPX* transcript accumulation and the significant increase in the *HvCAT* transcription level. On the contrary, the stress resulted in the significant decline in the *HvSOD* transcript accumulation in all analyzed genotypes. Under the stress conditions, accumulation of the *HvAPX* transcript was dependent on the BR biosynthesis and signaling, whereas accumulations of the *HvCAT* and *HvSOD* transcripts did not seem to be affected by the perturbations in the BR biosynthesis or signaling.

To put these results in a broader context, a meta-analysis of expression profiles of homologous genes in the model species *Arabidopsis thaliana* was performed based on the AtGenExpress transcriptome data accessed via the TAIR (www.arabidopsis.org) and the Arabidopsis eFP Browser 2.0 (http://bar.utoronto.ca/efp2/Arabidopsis/Arabidopsis_eFPBrowser2.html) databases. Expression profiles of the following Arabidopsis genes were analyzed: *APX1* (AT1G07890)**,** *APX2* (AT3G09640), *CAT1* (AT1G20630)**,** *CAT2* (AT4G35090), *SOD1* (AT1G08830) and *SOD2* (AT2G28190). The *APX1* gene is highly expressed in vegetative and generative tissues, and in reaction to drought the gene shows stable and relatively high expression level. The gene shows lower expression in the BR-deficient mutant *det* (deetiolated). This indicates that expression of this gene is BR-dependent. The *APX2* gene shows relatively low/moderate expression in vegetative tissues. During exposure to drought, the gene shows very low expression, which is not influenced by the stress. Expression of the *APX2* gene shows no reaction to the BR treatment. Thus, the *APX1* and *APX2* genes in Arabidopsis show diverse transcription profiles in the analyzed aspects. The *CAT1* gene shows very low expression level in all vegetative tissues. The gene expression is gradually increased in reaction to drought. Expression of this gene is not changed upon the BR treatment. The *CAT2* gene is highly expressed in bolts and leaves. During exposure to drought, the gene shows constant expression at the initial stage of stress, with the following decline and recovery phase. Similar to the *CAT1* gene, the *CAT2* expression is not changed upon the BR treatment. The *SOD1* gene is highly expressed in rosette and fully developed leaves. Interestingly, during drought, a decrease in the gene expression occurs and it remains very low. No change in expression of this gene in reaction to the BR treatment was reported. The *SOD2* gene shows high expression in rosette and fully developed leaves. Similar to the *SOD1* gene, a significant decrease in expression of the *SOD2* gene in reaction to drought was observed. Expression of the *SOD2* gene is not influenced in reaction to the BR treatment. Thus, expression of both *SOD1* and *SOD2* genes in Arabidopsis is not influenced by BR.

### 2.7. Profiles of the Enzymatic Antioxidant Activity under the Control and Drought Conditions

**Figure 7:**
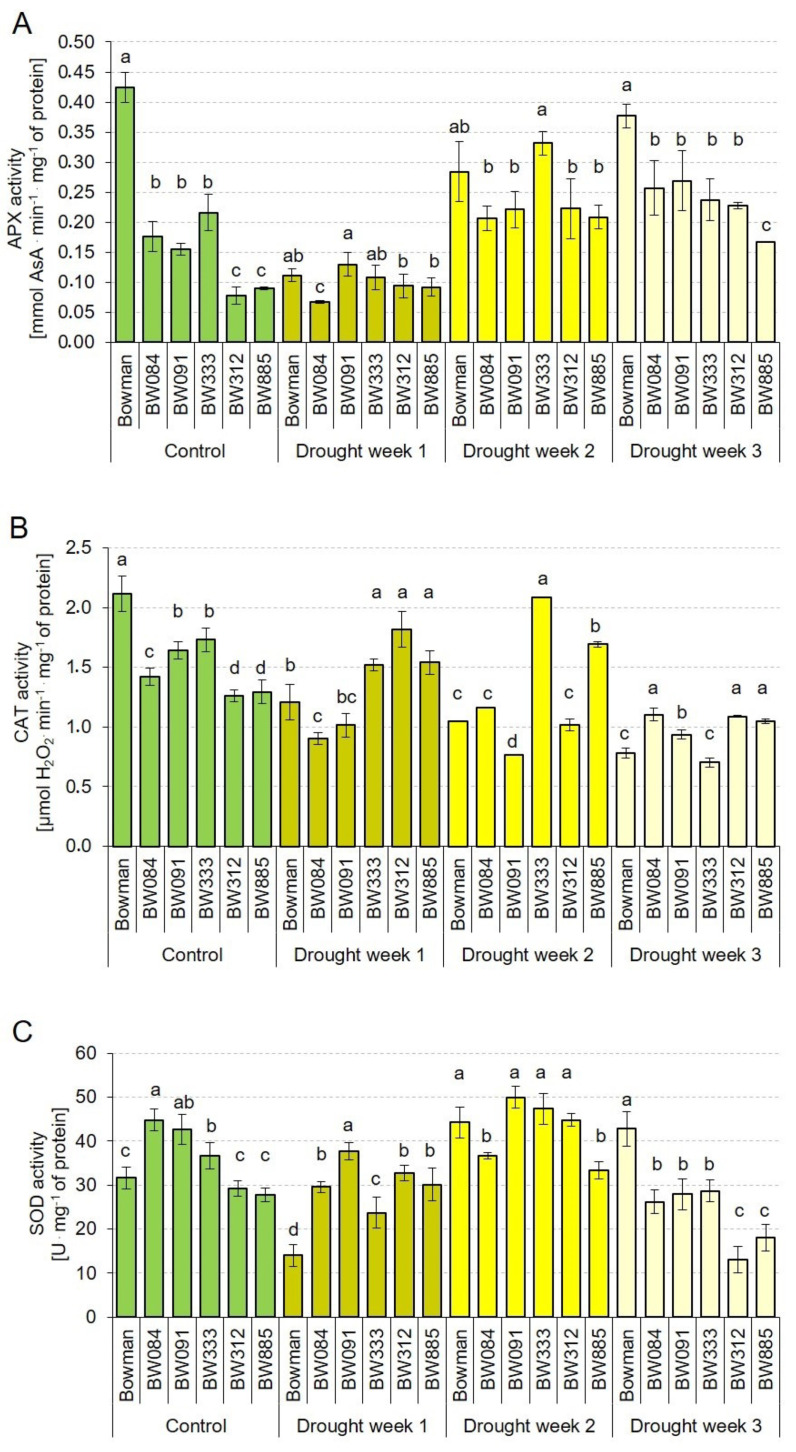
The profiles of the following enzymatic activities in the analyzed genotypes under the control conditions and during the three-week drought period: HvAPX (**A**); HvCAT (**B**); and HvSOD (**C**). *n*=3 replicates per genotype at each time point. The mean values are presented for each genotype, with error bars representing standard deviation. The mean values (±SD) marked with the same letters (separately for the control and drought-stressed plants) are not significantly different, according to the Duncan’s test (*p* ≤ 0.05). Details are given in the text.

As it is known that the transcription level of a gene is not always correlated with the activity of the encoded protein, in this study, activities of the antioxidant enzymes HvAPX, HvCAT and HvSOD were determined apart from the above-described analysis of the transcription profiles of the genes which encode these enzymes. To determine any time-dependent alteration in the profile of enzyme activities, analyses were performed under the control conditions and during three consecutive weeks of the increasing water deficit. Under the control conditions, the HvAPX activity was significantly decreased in the analyzed NILs when compared with the “Bowman” cultivar. The values reported in the NILs were from 18% (in BW312) to 50% (in BW333) of the reference value in the “Bowman” cultivar. Interestingly, within the group of NILs, two subgroups were identified: the BR-biosynthesis defective NILs (BW084, BW091 and BW333) showed significantly higher activities of HvAPX than the BR-insensitive NILs (BW312 and BW885) (Figure 7A). It indicates that defects in the BR signaling had more negative effect on the HvAPX activity than disturbances in the BR biosynthesis. It was quite surprising that the onset of drought stress (Drought Week 1) led to a decrease in the HvAPX activity in the majority of genotypes (except the BR-insensitive NILs, in which the activity remained at the respective control level). The most significant (ca. 4-fold) reduction of the APX activity was reported in the “Bowman” cultivar. Generally, during the first week of drought, the HvAPX activity was at a comparable level in all analyzed genotypes. During the second week of drought, the HvAPX activity increased significantly in all analyzed genotypes. Although some differences were reported among the genotypes, no tendency in the variation was detected. During the third week of water deficit, the highest level of HvAPX activity was reported in the “Bowman” cultivar. In the analyzed NILs, the values of APX activity was from 44% (in BW885) to 71% (in BW091) of the reference value reported in the “Bowman” cultivar (Figure 7A).

Interestingly, under the control conditions, profile of the HvCAT activity resembled the HvAPX activity profile, i.e., the highest HvCAT activity was reported in the “Bowman” cultivar and the values observed in the analyzed NILs were from 59% (in BW312) to 81% (in BW333) of the reference “Bowman” value. The group of NILs showed diverse profiles of the HvCAT activity: the BR biosynthesis-defective NILs (BW084, BW091 and BW333) showed higher activities of HvCAT than the BR-insensitive NILs (BW312 and BW885). Generally, during the drought period (Drought Weeks 1–3), the HvCAT activity in the analyzed NILs was at similar or higher level when compared with the “Bowman” value (Figure 7B). Thus, it may be concluded that the abnormalities in BR biosynthesis and signaling negatively affected the HvCAT activity under the control conditions; however, they had no significant effect on activity of this enzyme under the drought conditions.

As far as the HvSOD activity is concerned, under the control conditions, values of this parameter in the analyzed NILs were at a similar or higher level when compared with the “Bowman” cultivar. Interestingly, NILs deficient in the BR-biosynthesis (BW084, BW091 and BW333) showed significantly higher level of the HvSOD activity in comparison with the “Bowman” cultivar and the BR-insensitive NILs (Figure 7C). During the first week of drought, the lowest level of the HvSOD activity was reported in the “Bowman” cultivar, whereas, in the analyzed NILs, values of the HvSOD activity were in the range from 168% (in BW333) to 268% (in BW091) of the “Bowman” value. Interestingly, during the first week of drought, values of the HvSOD activity were decreased with respect to the control values in the “Bowman” cultivar and the BR biosynthesis-deficient NILs, but not in the BR-insensitive NILs, in which the HvSOD activity was at the control level. During the first week of drought, the most significant decrease in the HvSOD activity with respect to the control value was reported in the “Bowman” cultivar (decrease by 55%). During the second week of drought, the HvSOD activity was increased in all the analyzed genotypes; however, the degree of increase was different among the genotypes. Generally, during the second week of drought, no tendency in variation of this parameter among the genotypes was reported. During the third week of drought, significant differences in the HvSOD activity were observed between the “Bowman” cultivar and the analyzed NILs. The highest value of this parameter was reported in the “Bowman” cultivar (the value only slightly changed in comparison to the one reported in this genotype during the second week of drought). On the contrary, during the third week of drought, the HvSOD activity was significantly decreased in the BR biosynthesis-deficient and BR-insensitive NILs. Moreover, a significant difference in the HvSOD activity between the BR biosynthesis-deficient and BR-insensitive NILs was observed. The values of this parameter in the analyzed NILs were from 30% (in BW312) to 67% (in BW333) of the “Bowman” value (Figure 7C). Concluding, activities of the analyzed enzymatic antioxidants showed diverse profiles both under the control and drought conditions. Importantly, the obtained results point to a significant role of the BR biosynthesis and signaling in regulation of the HvAPX and HvCAT enzymatic activities under control conditions and the HvAPX and HvSOD activities during physiological reactions to prolonged (three-week) drought exposure.

### 2.8. Accumulation of Hydrogen Peroxide under the Control and Drought Conditions

**Figure 8:**
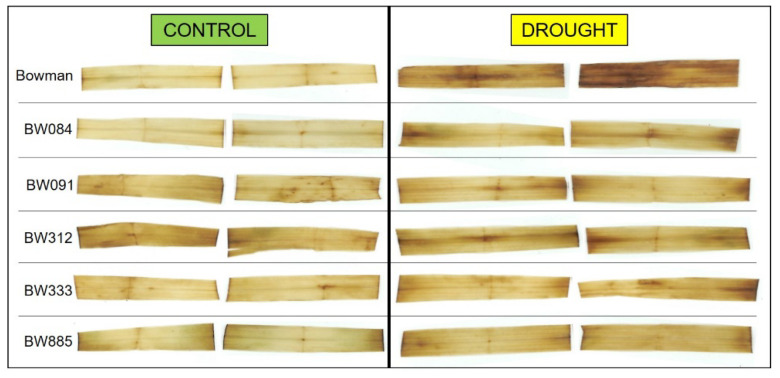
Accumulation of hydrogen peroxide in representative leaf fragments of each genotype grown under the control and drought conditions. The H_2_O_2_ accumulation is visible as brown spots.

In light of the above-described results, the next experiment was aimed at determining the hydrogen peroxide accumulation in leaves of the analyzed genotypes grown under the control and drought conditions. Comparison of the hydrogen peroxide accumulations among the analyzed genotypes and between the growth conditions led to a quite interesting observation. As expected, the drought stress led to an increase in the H_2_O_2_ accumulation in all analyzed genotypes. However, a noticeable difference was reported between the “Bowman” cultivar and the BR mutants. Under the drought stress conditions, leaves of the “Bowman” cultivar accumulated significantly more H_2_O_2_ than leaves of the BR mutants. Importantly, the drought-induced increase in the H_2_O_2_ accumulation with respect to the control conditions was most significant in the “Bowman” cultivar. This observation indicated that drought results in a more severe oxidative stress in plants of the “Bowman” cultivar than in the semi-dwarf BR mutants (Figure 8).

## 3. Discussion

During evolution, plants have developed a wide range of responses to drought, which are mostly represented by a variety of alterations in the growth rate and morphology of plants [8]. Therefore, in the analyzed NILs, the observed deficiency in the metabolism of BR as growth promoting hormone may be considered as a preadaptation to drought stress (on the level of physiological aspects and the favorable shoot/root biomass ratio). In fact, mutations identified in the analyzed NILs cause a semi-dwarf, erect architecture of the above-ground part of plants; however, they do not have any significant effect on root morphology or anatomy of the NILs [41]. It is known that the root-to-shoot biomass ratio is an important factor influencing drought tolerance. It is of significant importance for water use efficiency [4,30,31]. In this study, it was reported that the rate of growth of the semi-dwarf NILs under the control conditions, between Time Point 1 (optimal watering) and Time Point 4 (Control Week 3), was lower than that of the “Bowman” plants (Figure 1A). The water deficit significantly altered the dynamics of plant growth. Importantly, the rate of growth of the “Bowman” plants was particularly affected (Figure 1B). Based on the plant growth rate, and the comparison of plant height among the genotypes at the end of drought stress period (Time Point 4), it was concluded that plants of the “Bowman” cultivar were more negatively affected by the stress than plants of the semi-dwarf NILs. This conclusion is supported by the above-described difference in the H_2_O_2_ accumulation under the drought conditions between the “Bowman” cultivar and the semi-dwarf BR mutants. Importantly, in our previous experiment performed on two other semi-dwarf barley mutants, defective in the BR biosynthesis, in which missense substitutions were identified in the *HvDWARF* gene [71,72] and their reference cultivar “Delisa”, it was also reported that the growth rate and height of plants of the “Delisa” cultivar were more negatively affected by drought than that of the semi-dwarf mutants [32]. Therefore, it may be concluded that the enhanced tolerance to water deficit exhibited by the semi-dwarf, BR mutants of barley is independent of the genetic background of the mutants (it was observed in the mutants from the “Bowman” and “Delisa” backgrounds). Moreover, the enhanced tolerance to drought is displayed by the semi-dwarf mutants defective in the various genes encoding enzymes involved in the BR biosynthesis or signaling. These results reflect the observation that growth rate is one of the most drought-sensitive parameters [40,73]. The drought stress primarily reduces biomass growth and leaf expansion [4,8,73], therefore, the semi-dwarf NILs (with normal root structure) may be considered as preadapted to the stress [40]. Indeed, the root-to-shoot ratio was reported to increase continuously during plant exposure to drought [6]. Adjustment of metabolite accumulations is a secondary mechanism of adaptation to the stress conditions [70]. In our previous study on the BR-deficient semi-dwarf NILs, it was concluded that defects in the BR metabolism do not affect negatively their transpiration rate and stomatal conductance under the control conditions [31]. Similar results were obtained for other BR-deficient mutants of barley and rice [30,32]. In the same experiment, it was also indicated that, in reaction to drought, the semi-dwarf NILs maintained the transpiration rate and stomatal conductance at a similar or even higher level when compared with the “Bowman” cultivar [31]. This result is quite intriguing considering the enhanced tolerance to water deficit exhibited by the semi-dwarf, BR-deficient NILs. Therefore, in this study, the analysis of changes in the relative water content in leaves of the genotypes was performed during the three-week period of increasing water deficit. As expected, the increasing water deficit resulted in a decrease of the relative water contents in leaves of the genotypes. However, the decline in the relative water content was the steepest in the “Bowman” cultivar. Upon the three weeks of water deficit (Time Point 4), the relative water contents reported in the analyzed semi-dwarf NILs were significantly higher than in the “Bowman” cultivar (Figure 2). The results were validated by the analysis of leaf reflectance parameters (the WBI values.) The decrease in the water content in leaf tissues is associated with a decrease in the WBI parameter value [74]. Generally, higher WBI values in the NILs (in comparison to the “Bowman” cultivar) after the three weeks of drought indicate their better adaptation to the water scarcity (Figure 3A). This is probably the result of less water demand or better redistribution of water within the semi-dwarf plants under the stress conditions. Indeed, drought-tolerant genotypes typically show higher water retention [6]. Taking the above-described results into account, we postulate that the previous observation indicating that, under drought stress, the semi-dwarf NILs maintained the transpiration rate and stomatal conductance at a similar or even higher level when compared with the “Bowman” cultivar [31] stems from the fact that the relative water contents reported in the analyzed semi-dwarf NILs were significantly higher than in the “Bowman” cultivar. In fact, a modulation of stomatal conductance without complete stomatal closure is characteristic strategy of drought-adapted plants. It should also be mentioned that stomatal closure and subsequent limitation on the CO_2_ assimilation lead to the ROS production [39,43]. These observations point to a conclusion that the enhanced tolerance to water deficit exhibited by the semi-dwarf mutants of barley results primarily from their favorable shoot/root biomass ratio, which is the consequence of perturbations in BR biosynthesis and signaling.

Since, under the drought conditions, the semi-dwarf NILs maintained the transpiration rate and stomatal conductance at a similar or even higher level when compared with the “Bowman” cultivar, in the next step of the study, we aimed to determine the RuBisCO activity in the analyzed genotypes under the control and drought conditions. Under the control conditions, the RuBisCO activity was significantly higher in the “Bowman” cultivar when compared with the BR-deficient NILs (Figure 4). This result was not unexpected, as it was reported that BRs have a stimulatory effect on the CO_2_ assimilation and the RuBisCO activity. In rice, plants with increased BR contents showed a higher efficiency of CO_2_ assimilation [75]. On the other hand, an application of BR biosynthesis inhibitor in cucumber plants reduced the RuBisCO activity [53,54]. However, the analysis of drought influence on the RuBisCO activity in the analyzed genotypes brought some interesting results. The stress resulted in a very significant decline in the RuBisCO activity specifically in the “Bowman” cultivar. On the contrary, drought did not lead to a significant alteration in the RuBisCO activity in the analyzed NILs. Similar intriguing results were obtained in our previous experiment on the semi-dwarf barley mutants (in the *HvDWARF* gene) and their reference cultivar “Delisa”. Under the control conditions, the RuBisCO activities in the mutants were significantly lower than in the “Delisa” cultivar. However, the drought-related decline in the RuBisCO activity was the most prominent in the “Delisa” cultivar. Consequently, values of the RuBisCO activity under the stress conditions were at a similar level in all analyzed genotypes [32]. Therefore, it may be concluded that the RuBisCO activity is much less affected by the drought stress in the semi-dwarf, barley BR mutants in comparison with their reference cultivars. This phenomenon seems to be independent of the genetic background of the mutants, as it was observed in the mutants from the “Bowman” (this study) and “Delisa” backgrounds [32]. Moreover, this phenomenon may indirectly illustrate the enhanced tolerance to water deficit exhibited by the semi-dwarf, BR mutants of barley, as they maintain stomatal conductance (and CO_2_ supply) at a similar or even higher level when compared with the “Bowman” cultivar. 

The profile of RuBisCO activity in the analyzed genotypes under the control and drought conditions was a premise for determining the soluble sugar contents. Abnormalities in BR biosynthesis or signaling negatively affect accumulation of glucose and fructose under the control conditions. The lower accumulations of glucose and fructose reported in the analyzed NILs may indirectly reflect the above-mentioned reduced values of the RuBisCO activity which were observed in the mutants under the control conditions [55]. The drought stress resulted in the significant (at least two-fold) increase in the accumulations of these monosaccharides in all analyzed genotypes, however, the highest contents of these compounds were reported in the “Bowman” cultivar (Figure 5A,B). The results indicate that perturbations in the BR metabolism negatively influence the glucose and fructose accumulation in a similar manner both under the control and stress conditions. In line with this observation, in a tomato (*Lycopersicon peruvianum*) mutant with nonfunctional DWARF enzyme (involved in BR biosynthesis), the sugar content was also reduced [76]. Interestingly, high sugar content was reported to repress the activity of another BR biosynthetic enzyme—CPD [77]. It should be kept in mind that sugars play a role as signaling molecules which promote plant cell division and differentiation [68]. Interaction between the BR and glucose signaling pathways seems to be mutual, as it was recently reported that glucose influences interaction between the BRI1 receptor kinase and the BAK1 co-receptor in a concentration-dependent manner. Moreover, the BRI1 and BAK1 kinases participate in regulation of the sugar responses [65]. It is known that glucose enhances expression of the *BZR1* gene, which encodes a key transcription factor regulating the BR-dependent gene expression. Moreover, glucose influences expression of the majority of the BR-regulated genes, and 58% and 42% of them are regulated synergistically and antagonistically, respectively [66,68]. It was suggested that the glucose and BR signaling pathways act antagonistically at low glucose concentration and synergistically at higher glucose concentrations [66]. Nevertheless, it should be kept in mind that interaction between the glucose and BR signaling pathways is still poorly understood [68,78]. In this study, it was found that drought stimulates the accumulation of both monosaccharide sugars, and this physiological reaction to the stress is maintained in the analyzed NILs. This drought-stimulated increase in the accumulation may reflect the fact that glucose and BR coregulate a large number of genes involved in abiotic stress responses [66]. In our previous study, it was reported that, in reaction to drought, the endogenous CS (biologically active form of BRs) accumulation increased significantly in the analyzed NILs and the “Bowman” cultivar [31]. As far as the sucrose accumulation is concerned, under the control conditions, the highest content of this disaccharide was reported in the “Bowman” cultivar (Figure 5C). Under the control conditions, the genotype-dependent profile of sucrose accumulation was similar to the glucose and fructose accumulation profiles (although the sucrose content was several times higher than the contents of glucose and fructose). Therefore, it may be inferred that the reduced content of sucrose in the analyzed NILs may be correlated with the lower accumulations of glucose and fructose as they are substrates of the sucrose biosynthesis. Thus, it may be concluded that the reduced content of sucrose in the mutants may be an indirect consequence of the perturbations in BR biosynthesis or signaling [79]. In contrast to the pattern of changes (significant increase) in the glucose and fructose accumulation in reaction to drought, the stress conditions induced a significant reduction in the sucrose content in all the analyzed genotypes (Figure 5C). Profile of changes in the sucrose content during reaction to the water deficit seems to be different between plant species. In Arabidopsis, the sucrose accumulation increased in reaction to drought [80,81], whereas, in rice, the sucrose content decreased under the water-deficit stress [82]. It was also reported in maize that vacuolar invertase is induced by drought [58]. Thus, the pattern of drought-induced changes in the sucrose content that was found in this study in barley is in line with the profile reported in other monocots—rice and maize. It is an open question whether this inter-species difference represents a more general difference between dicots and monocots in this aspect of physiological reaction to drought. Taking into account that, under the stress conditions, the lowest content of sucrose was reported in the “Bowman” cultivar, and that this pattern is opposite to the profiles of glucose and fructose accumulation in this genotype, it may be inferred that the significant increase in glucose and fructose accumulation in reaction to drought occurs at the expense of sucrose accumulation. In this case, glucose and fructose would be accumulated as products of the sucrose decomposition, which may proceed in the cytosol and/or in phloem tissue [78]. It should also be kept in mind that the lowest content of sucrose reported in the “Bowman” cultivar may ultimately lead to impairment of export rate from source to sink [58]. As far as raffinose is concerned, under the control conditions, its accumulation in the analyzed NILs was at a similar or higher level when compared with the “Bowman” value. Although no general tendency in the drought-induced alterations in the raffinose accumulation could be determined, under the stress conditions, the lowest content of this compound was reported in the “Bowman” cultivar. The lowest content of raffinose in the “Bowman” cultivar under the stress conditions may result from the fact that raffinose is synthesized from sucrose [83], and the lowest content of sucrose was reported in the “Bowman” cultivar. It is known that, in comparison with other osmoprotectants, sugars have the greatest contribution to maintaining osmotic potential under stress conditions. Soluble sugars also play a significant role in the ROS scavenging [62,63]. During drought stress, the contents of various sugars (particularly, glucose, fructose and raffinose) usually increase despite the reduction in the CO_2_ assimilation [83,84]. Moreover, these carbohydrates accumulate earlier than other metabolites during plant reaction to drought [70]. This study found that there are significant differences in the accumulation profiles of glucose, fructose (significant increase in the contents of these monosaccharides), sucrose (decrease in accumulation of this disaccharide) and raffinose (no significant change in the content) during reaction of the barley plants to drought. Thus, the obtained results offer more detailed insights into the sugar metabolism in barley under the control and drought conditions and provide evidence for the role of BR in regulation of the sugar metabolism.

Analysis of the relative transcription level of the *HvAPX, HvCAT* and *HvSOD* genes encoding key enzymatic antioxidants provided some interesting results. Firstly, disturbances in the BR biosynthesis and signaling negatively affect the relative transcription levels of the *HvAPX* and *HvSOD* genes under the control conditions (Figure 6A,C). Under the control conditions, transcription of the *HvCAT* gene was at the relatively very low level in all analyzed genotypes. Despite the fact that in the NILs the drought-induced increase in the *HvAPX* transcription was much more significant than in “Bowman”, under the stress conditions, the values observed in the mutants were significantly lower than in the “Bowman” cultivar (Figure 6A). The results indicate that the abnormalities in BR biosynthesis and signaling negatively affect accumulation of the *HvAPX* transcript both under the control and drought conditions. Transcription of the *HvCAT* gene in barley is significantly stimulated by drought, but the abnormalities in BR biosynthesis and signaling do not seem to influence the profile of *HvCAT* transcript accumulation under the stress conditions. Surprisingly, drought led to a significant decline in the *HvSOD* transcript accumulation in all analyzed genotypes. The abnormalities in the BR metabolism do not influence on the *HvSOD* gene transcription under the stress conditions. The relative transcription levels indicated that each of the analyzed genes displays a distinct profile of transcript accumulation in the analyzed genotypes under both growth conditions. In our previous experiments it was reported that BRs regulate the non-enzymatic antioxidant homeostasis, particularly through modulation of the ascorbate, glutathione and tocopherols (α-tocopherol and γ-tocopherol) accumulation under the control and drought conditions. It was also reported that the drought-stimulated increase in the accumulation of this antioxidant triad occurs in a coordinated manner [42]. The study presented here showed that the profiles of the *HvAPX, HvCAT* and *HvSOD* transcript accumulation have more differential patterns. The differential patterns of the *HvAPX, HvCAT* and *HvSOD* transcript accumulations were also reported in other barley genotypes [51]. However, the transcription profiles of the antioxidant genes may also show different patterns depending on various genotypes of the same species and magnitudes of the stress [6,85]. 

The conducted meta-analysis of expression profiles of the Arabidopsis genes encoding the enzymatic antioxidants allowed for comparison with the results obtained in this study. The comparison provided insights into similarities and differences in the regulatory mechanisms between the model dicot species and barley, which is an important monocot crop. As far as similarities are concerned, transcription levels of the *HvAPX* gene in barley and the *APX1* gene in Arabidopsis are BR-dependent. Transcription of the *HvCAT* gene and the *CAT1* gene in Arabidopsis is at a relatively very low level under the control conditions. Transcription of the *HvCAT* gene and the Arabidopsis *CAT1* gene is significantly increased in reaction to drought. Another interesting similarity is that expression of the of *HvCAT* gene as well as the Arabidopsis *CAT1* and *CAT2* genes is not influenced by BR. An important and intriguing similarity in the regulatory mechanisms is that drought leads to the significant decline in expression of the *HvSOD* gene in barley and the *SOD1* and *SOD2* genes in Arabidopsis. The major difference between the regulatory mechanisms in these species is related with the role of BR: perturbations in the BR biosynthesis or signaling negatively affect the *HvSOD* transcript accumulation under the control conditions in barley, whereas expression of both *SOD1* and *SOD2* genes in Arabidopsis is not influenced by BR.

Noteworthy, the differential expressions of the *APX, CAT* and *SOD* genes are frequently not correlated with changes in activities of the encoded enzymes [51]. This difference may result from the fact that transcript synthesis is regulated at a single level of transcription efficiency, whereas activity of the encoded protein may be potentially regulated at several steps: translation (protein production) efficiency, biochemical modulation of the enzyme activity (which may be mediated by accumulation of various metabolites, including substrate and product of the reaction catalyzed by the enzyme) and ultimately at the protein degradation step. Thus, the multiple steps of protein activity modulation enable influence of numerous factors. Although we recently gained insights into mechanisms of the BR-regulated genes expression [12,15], a role of BRs in regulation of protein activity and turnover is still far from being elucidated.

In our previous experiments, it was reported that, in the analyzed NILs, the accumulation of reduced form of ascorbate (AsA) was reduced to a various degree under both the control and drought conditions when compared with the “Bowman” value. The results indicate that endogenous BRs regulate the AsA homeostasis under both conditions [42]. The reduced form of ascorbate is a crucial antioxidant substrate (electron donor) in the reaction of H_2_O_2_ scavenging, which is catalyzed by the APX enzyme [6,44]. This study found that that, under the control conditions, the HvAPX activity was significantly decreased in the analyzed NILs when compared with the “Bowman” cultivar. Therefore, we postulate that the reduced HvAPX activity results from the decreased accumulation of AsA as the reaction substrate. This hypothesis seems to be validated by the observation that, during the third week of drought, the highest level of HvAPX activity was reported in the “Bowman” cultivar, whereas it was reduced in the BR mutants. This observation corresponds with the decreased accumulation of AsA in the mutants. The differences in the AsA accumulation under the drought conditions reported between the “Bowman” cultivar and the analyzed NILs may result from a lower recycling rate of AsA from DHA (the DHA concentrations were significantly higher in the drought-stressed mutants). The lower rate of AsA regeneration is caused by the reduced accumulations of glutathione and tocopherols in the drought-stressed NILs [42], as it is known that glutathione and tocopherols are important components of the antioxidant regeneration system involved in the recycling of AsA from DHA [43,44,86,87]. The HvAPX activity in the majority of genotypes was decreased during Drought Week 1. It is suggested that the APX enzyme functions as a fine regulator of steady-state accumulation of intracellular ROS as signaling molecules, whereas CAT functions as a bulk remover of excess ROS produced during stress conditions [43]. Thus, an explanation for the initial decrease in the HvAPX activity at the onset of drought might be that preliminary physiological reaction of plants to the stress results in the reduction of activity of the APX enzyme, which functions to balance the ROS homeostasis, in order to initiate an adaptive switch to efficient ROS scavenging. It should also be mentioned that a decrease in the APX activity in reaction to drought was also reported in other crop species, including rice [43], and that this reaction is highly dependent on plant species and magnitude of stress [4]. The HvAPX activity increased gradually during exposition of plants to water deficit from the first to third week of drought. It is consistent with results reported in other experiments [6,33,44]. Generally, exogenous applications of BR in experiments conducted on various plants species led to increase in activities of the antioxidant enzymes, however, the reactions were species-dependent [19,88,89]. Noteworthy, over the years some contradictory results concerning influence of drought stress on activities of the antioxidant enzymes have been gathered and no general pattern of the enzymes’ activities in reaction to the stress could be outlined [43,51].

This study found that that the abnormalities in BR metabolism negatively affect the HvCAT activity under the control conditions; however, they have no significant effect on activity of this enzyme under the drought conditions. The catalase activity was also reported to be differentially regulated (from a decrease up to an increase) in reaction to drought, and this differential pattern of changes was dependent on plant species [43]. Moreover, activities of these enzymes may significantly change in time during prolonged exposure to the stress [6]. Another level of complexity constitutes a diverse regulation at the transcriptional and posttranslational levels. In wheat (*Triticum aestivum*), severe drought was reported to stimulate the catalase activity, but at the transcriptional level, no stimulatory effect was observed [90]. These results indicate a complex mechanism of the catalase activity regulation. It was suggested that the catalase activity is stimulated under the severe drought conditions, whereas, under moderate water deficit, H_2_O_2_ scavenging is mediated by ascorbate peroxidase. Thus, it was postulated that, under the severe drought conditions, the major role of catalase is to maintain the ascorbate peroxidase activity [43]. 

Activity of the third crucial component of the enzymatic antioxidant machinery, SOD, was reported to be regulated by soluble sugars [60,61]. Interestingly, the BR-deficient mutant *det2* (defective in the BR biosynthesis) in Arabidopsis showed an enhanced oxidative stress tolerance which was correlated with a constitutive increase in the SOD activity [91]. In the study presented here, a similar observation was made for the BR-deficient NILs BW084, BW091 and BW333 carrying mutations in the barley BR biosynthetic genes *HvCPD*, *HvBRD* and *HvDIM,* respectively. Under the control conditions, in the BR-deficient NILs, the HvSOD activity was significantly higher than in the “Bowman” cultivar. Interestingly, in the BR-insensitive NILs (BW312 and BW885), the HvSOD activity was similar to the “Bowman” value (Figure 7C). These results validate the hypothesis that, under the control conditions, endogenous BRs may repress the activities of the stress response-related proteins to ensure normal growth and development. However, it is not completely understood whether BRs exert this effect directly or indirectly [19,91]. Taking into account the HvSOD activity in the BR-insensitive NILs, it may also be postulated that the BR-dependent negative regulation of the HvSOD activity can be mediated by a signaling pathway which is alternative to the canonical BRI1-BAK1 pathway (which is defective in the analyzed NILs), i.e., by the BR signaling pathway initiated by the heterotrimeric G proteins [92].

## 4. Materials and Methods 

### 4.1. Plant Material 

In this study, experiments were performed on a group of semi-dwarf barley (*Hordeum vulgare*) Near-Isogenic Lines (NILs) and the reference barley cultivar “Bowman”. The NILs represent the semi-dwarf barley mutants which were previously characterized genetically and physiologically as displaying defects in the BR biosynthesis or signaling [41]. The NILs were developed by recurrent crossing of the original mutants into the genetic background of the “Bowman” cultivar [93]. Importantly, owing to this approach, each of the NILs harbors a specific and mapped genomic introgression region, which is derived from the original mutant, in the homogeneous genetic background of the “Bowman” cultivar. The homogenous genetic “Bowman” background is shared by all the NILs [31]. Owing to the homogenous genetic background of all tested genotypes, the use of NILs simplifies comparative physiological analyses and interpretations [94].

The plant material of these experiments included NILs showing defects in the BR biosynthesis: BW084 (*brh13.p*) carrying a missense mutation in the *HvCPD* gene; BW091 (*brh3.g*), in which a nonsense mutation in the *HvBRD* gene was identified; and BW333 (*ert-zd.159*), which harbors a missense mutation in the *HvDIM* gene. Additionally, the material included NILs characterized by abnormalities in the BR perception: BW312 (*ert-ii.79*) and BW885 (*uzu1.a*), which carry missense mutations in different domains of the HvBRI1 receptor. Details on the identified mutations were published in our previous publication [41]. Importantly, the abnormalities in BR metabolism result in phenotypic alterations only in the above-ground part of the mutants, causing the semi-dwarf stature. However, the mutations have no significant effect on the root anatomy and architecture [41]. Therefore, the selected NILs constitute an ideal material for the research on reaction of the semi-dwarf BR mutants to drought [31].

### 4.2. Plant Growth Conditions and Experimental Design

A detailed description of preparation of soil mixture for plant cultivation and calculations of the maximum (100%) soil water capacity were published in our previous study [32]. Protocol and conditions of seed germination on Petri dishes and transfer of 4-day-old seedlings to pots containing the soil mixture were also described by us previously [31]. Pots with seedlings were transferred to a growth chamber in which the conditions of cultivation were the following: 12-h photoperiod (throughout the whole experiment), light intensity (250 μmolm^−2^ s^−1^), temperature on Days 4–7 of the experiment was 23 °C (day and night) and temperature on Days 8–28 of the experiment was 18°/15 °C (day/night). All plants of each genotype (the “Bowman” cultivar and NILs) were watered optimally (70% of soil water capacity) until the 28th day of the experiment. On the 29th day of the experiment, plants of each genotype were divided into two groups and the temperature in growth chamber was set at 22°/18 °C (day/night), 12-h photoperiod. The first group of plants was watered optimally until the end of the experiment (50th day of vegetation). The plants from the second group were watered optimally for the last time on the 29th day of vegetation, and then the plants were grown under increasing water deficit due to watering cessation. The soil water content was monitored in every pot on each day of the experiment and the only water supplementation was performed to balance an uneven water loss between the particular pots, if one occurred. At the beginning of the second week, from the moment of watering cessation, the soil water content reached 25% and this level of drought was maintained to the end of experiment (the 50th day of vegetation). Thus, plants of the second group were exposed to water deficit for three weeks. More technical details of setting and maintaining the soil water content were published in our previous study [32]. 

For all observations/measurements, the starting point was the 29th day of vegetation (optimally watered plants, marked on all figures as “control”). Plant height was measured for the first time on the 29th day of vegetation (optimal watering). Next, measurements were performed after the first, second and third week of drought stress (described in figures as “Drought Week 1”, “Drought Week 2” and “Drought Week 3”) and also at the corresponding time points in plants from the first, optimally watered group (marked in Figure 1A as “Control Week 1”, “Control Week 2” and “Control Week 3”). Leaf water content and leaf reflectance were measured on the 29th day of vegetation (optimal watering, control) and in the group of plants exposed to drought (after the first, second and third weeks of drought stress).

### 4.3. Plant Growth, Changes in Leaf Water Content and Observations of Plant Wilting 

Plant growth rate was expressed as changes in plant height (length of the aerial part of plants). Leaf water content was measured in the third leaf. The third leaves were collected from 12 plants of each genotype, weighed separately to obtain the fresh mass and then dried for 48 h (105 °C) to obtain the dry mass. Leaf water content was calculated as percent of water in the fresh mass. Observations of plant wilting symptoms were made visually and described. 

### 4.4. Analysis of Reflectance Parameters

The reflectance spectrum of the light radiation in the range 400–1000 nm was measured using the CID Bio-Science CI-710 spectrometer (USA) with SpectraSnap software. The reflectance was measured on the upper (adaxial) surface of the third leaf. For each genotype, the measurement was performed in 7 replicates under both conditions. Reflectance factors were calculated on the basis of reflectance spectra, which allowed determining changes in the water content (WBI) in the leaves, the carotenoid pigments content (CRI1) and concentration ratio of carotenoids to chlorophyll (SIPI). The PRI index, indicative of light use efficiency by the leaves in the PAR range, was also calculated.

WBI = R_900_ R_970_^−1^ [74]
CRI1 = (R_520_^−1^ − R_550_^−1^) R_800_ [95]
SIPI = (R_800_ − R_445_) (R_800_ + R_680_)^−1^ [96]
PRI *=* (R_570_ − R_531_) (R_570_ + R_531_)^−1^ [97]

where R denotes the reflectance intensity at the radiation wavelength given in the subscript.

### 4.5. Determining Ribulose-1,5-Bisphosphate Carboxylase/Oxygenase (RuBisCO) (EC 4.1.1.39) Activity 

The RuBisCO activity was assayed according to the method described elsewhere [98]. In this method, the enzyme activity is coupled to the NADH oxidation using 3-phosphoglycerate (PGA) kinase and glyceraldehyde 3-phosphate (GAP) dehydrogenase. The oxidation of NADH was continuously monitored in spectrophotometer. The leaf samples were collected from 8 plants/genotype/growth condition (the third leaf of each plant was collected), and their area was measured using Leaf Area Meter Cl-202, CID Bio-Science, USA. Samples were immediately frozen in liquid nitrogen and homogenized with insoluble 1% PVPP and extraction buffer [100 mM Bicine pH 7.8, 1 mM EDTA, 5 mM MgCl_2_, 5 mM DTT, 0.002% BSA (*w/v*)]. The reaction was initiated by adding leaf extract to the assay buffer containing 50 mM Bicine pH 8.0, 1 mM EDTA, 18.5 mM NaCl, 15 mM MgCl_2_, 9.2 mM DTT, 0.6 mM RuBP, 9.2 mM NaHCO_3_, 0.4 mM NADH, 0.5 mM ATP, 4.6 mM phosphocreatine, 1.3 U of phosphocreatine kinase, 47 U of phosphoglycerate kinase and glyceraldehyde 3-phosphate dehydrogenase. The absorbance was monitored for 2 min at 340 nm. The determination of RuBisCO activity under both conditions (control and drought) was performed in three replicates per genotype (three independent samples collected from different plants of each genotype). Each replicate was assayed three times. All enzymatic measurements were performed using a Ultrospec 2100 pro (Biosciences Amersham, Sweden) spectrophotometer. The chemicals were purchased from Sigma-Aldrich (Poznan, Poland).

### 4.6. Soluble Sugar Content Analysis

Analysis of soluble sugars was performed according to modified protocols described elsewhere [32,99]. Briefly, the third and fourth leaves were collected from 5 plants of each genotype grown under the control and drought conditions. Subsequently, the leaf samples (0.3 g F.W.) were lyophilized and ground. Samples (5 mg D.W.) were extracted with 1 mL of deionized water, diluted with acetonitrile 1:1 (*v*/*v*) and centrifuged (15 min, 2200× *g*, Universal 32R, Hettich, Germany). The supernatant was filtered through a 0.22-μm membrane filter (Costar Spin-X, Corning, NY, USA). The sugar content analysis was performed using the Agilent 1200 binary system (Agilent Technologies, Germany) coupled with ESA Coulochem II detector (ESA, USA). Separation was achieved on Hamilton RCX-10 7 µm; 250 × 4.1 mm column (Hamilton, USA) at flow rate: 1.5 mL/min, in gradient mode of 70 mM aqueous NaOH solution vs. 500 mM NaCH_3_COO in 70 mM NaOH. Pulsed amperometric detection on the gold electrode was applied (analytical potential 200 mV; oxidizing potential 800 mV, reducing potential –900 mV, with reference to a palladium electrode). Contents of the following soluble sugars were determined: glucose, fructose, sucrose and raffinose. All analyses were performed in four replicates per genotype/growth condition.

### 4.7. Analysis of the HvAPX, HvCAT2 and HvSOD1 Transcript Accumulation

Quantitative Real Time PCR analysis of the *HvAPX* (GenBank Acc. No. AJ006358.1), *HvCAT2* (GenBank Acc. No. U20778.1), and *HvSOD1* (GenBank Acc. No. HM537232.1) transcripts’ accumulations was performed using the 7500 Real Time PCR System (Applied Biosystems, Foster City, CA, USA). Upon sampling, 0.05 mg of tissue was used for RNA extraction from the second leaves of control plants of each genotype and from the third leaves of drought-stressed plants of each genotype. In the drought-stressed plants, leaf tissue was collected from the third leaves upon the three-week period of water shortage because the second leaves were at that time completely dried in plants of the reference cultivar “Bowman”. Leaf tissue sampling for the RNA extraction was performed in three replicates per genotype (three independent samples collected from different plants of each genotype) under both conditions (control and drought). After the sampling, the leaf tissue was immediately frozen in liquid nitrogen. The RNeasy Plant Mini Kit (Qiagen, Hilden, Germany) was used for RNA extraction according to the manufacturer’s protocol. Upon the extraction, 500 ng of RNA were added to each reverse transcription reaction which was combined with the genomic DNA contamination Wipeout Buffer (QuantiTect Reverse Transcription Kit, Qiagen, Hilden, Germany) according to the manufacturer’s protocol in order to avoid any unspecific amplification of genomic DNA template. RNA and cDNA concentration and quality were determined spectrophotometrically (UV-vis Spectrophotometer, Quawell, San Jose, CA, USA). The qPCR primers and TaqMan MGB probes were designed using the Primer Express Software v 3.0.1 (Applied Biosystems by Life Technologies, Foster City, CA, USA), and their sequences are given in Supplementary Materials (Table S1). The qPCR amplifications for the target genes and the endogenous control *HvADP* gene (GenBank Acc. No. AJ508228.2) were conducted in triplicates as described elsewhere [100]. The qPCR data were analyzed using the 7500 real time PCR Sequence Detection Software v1.3. Relative standard curve method (Applied Biosystems) was employed for relative gene expression calculations.

### 4.8. Assay of the Ascorbate Peroxidase (APX) (E.C.1.11.1.11) Activity 

The ascorbate peroxidase activity was determined according to the method published elsewhere [101]. Leaf samples, collected from the second (in the control plants) and third leaves (in the drought-stressed plants), were homogenized at 4 °C with 50 mM phosphate buffer (pH 7.0) containing 1 mM EDTA and 0.5 mM ascorbic acid, and centrifuged at 16,000× *g*. The reaction mixture containing the phosphate buffer (50 mM, pH 7.0), 0.5 mM ascorbic acid, and supernatant was incubated in the dark for 3 min. Then, 15 mM H_2_O_2_ was added. The oxidation of ascorbic acid was monitored in a quartz cuvette at 290 nm. The respective control reaction mixtures contained buffer instead of the H_2_O_2_ solution. Activity of the APX enzyme was expressed as μmol AsA oxidized per minute per mg of the protein. The determination of the APX activity under both conditions (control and drought) was performed in three replicates per genotype (three independent samples collected from different plants of each genotype). Each replicate was assayed three times.

### 4.9. Assay of the Catalase (CAT) (EC 1.11.1.6) Activity

The catalase activity was assayed according to the protocol published elsewhere [102]. Leaf samples, collected from the second (in the control plants) and third leaves (in the drought-stressed plants), were homogenized at 4 °C with 50 mM phosphate buffer (pH 7.5), 1 mM EDTA, and centrifuged at 16,000× *g*. The CAT activity was assayed in a reaction mixture composed of 50 mM phosphate buffer (pH 7.5) and 15 mM H_2_O_2._ The reaction was started after adding 200 μL of supernatant to the reaction mixture. The CAT activity was measured as a decrease in absorbance at 240 nm, resulting from the H_2_O_2_ decomposition. A decrease in absorbance of 0.0145 corresponded with 1 μM H_2_O_2_ decomposed by CAT. Activity of the enzyme was expressed as μmol H_2_O_2_ decomposed per minute per mg of the protein. The determination of CAT activity under both conditions (control and drought) was performed in three replicates per genotype (three independent samples collected from different plants of each genotype). Each replicate was assayed three times.

### 4.10. Assay of the Superoxide Dismutase (SOD) (E.C.1.15.1.1) Activity

The activity of superoxide dismutase was assayed according to a protocol described elsewhere [103]. Leaf samples, collected from the second (in the control plants) and third leaves (in the drought-stressed plants), were homogenized at 4 °C with 50 mM phosphate buffer (pH 7.8) with addition 1% PVPP and centrifuged at 16,000× *g*. The reaction mixture contained 50 mM potassium phosphate (pH 7.8), 1 mM EDTA, 1 unit of SOD, 56 mM nitroblue-tetrazolium (NBT), 0.1 mM xantine, 0.03 units of xantine oxidase and the supernatant. The absorbance was monitored at 560 nm. The inhibition percentage of the NBT reduction is a measure of the SOD activity. One unit of SOD is the amount of extract that provides half-maximum inhibition. The SOD activity determination under both conditions (control and drought) was performed in three replicates per genotype (three independent samples collected from different plants of each genotype). Each replicate was assayed three times.

### 4.11. Determining Protein Content

Protein content was determined according to the method described elsewhere [104], with bovine serum albumin as a calibration standard.

### 4.12. Determining the Hydrogen Peroxide Accumulation

Accumulation of hydrogen peroxide was determined in leaves histochemically using 3,3′-diaminobenzidine (DAB) according to method described elsewhere [105] with a slight modification. In this method DAB forms an insoluble brown polymer in the presence of H_2_O_2_ and peroxidase. Leaf fragments from five plants of each genotype grown under the control and drought conditions were vacuum-infiltrated for 15 min in a solution containing DAB (Sigma D-8001) dissolved in 0.1 M HCl (pH 3.8) under dim light. After the infiltration, the leaf fragments were left in the solution for 1 h in the daylight. Chlorophyll was removed from the leaf fragments using 80% ethanol at 60 °C (the leaf fragments were incubated in ethanol until all chlorophyll was removed). The hydrogen peroxide accumulation was visible as brown spots.

### 4.13. Statistical Analysis

Representation of individual plants of each genotype within each sample and the number of replicates performed for each analysis/measurement are described above. Statistical differences were calculated based on the Duncan test (*p* ≤ 0.05) with use of the Statistica program. The statistical analyses were performed separately for the optimally watered plants (control) and the drought-stressed plants (comparison of the averages obtained in a particular analysis for the “Bowman” cultivar and the analyzed NILs within the control and drought-stressed group). The mean values are presented in the figures together with standard deviations and letters informing about the statistical significance of the reported differences.

## 5. Conclusions

**Figure 9:**
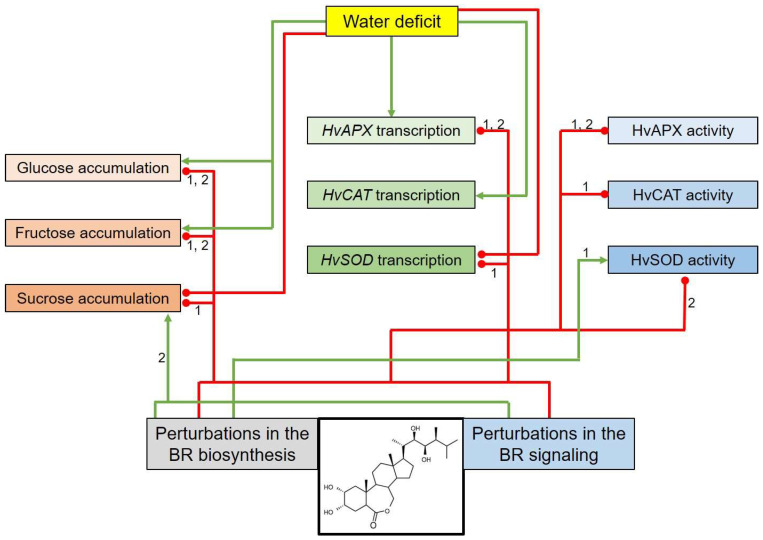
The major conclusions of the study provide insights into metabolic mechanisms of drought response in barley and the role of endogenous BRs in regulation of these processes. Green arrows indicate stimulation, whereas red lines with the bullet points represent suppression: 1, under the control conditions; and 2, after the three-week drought stress. Detailed description is given in the text.

The conducted multidirectional analyses allowed insights into the key metabolic mechanisms of drought response in the semi-dwarf barley mutants and the role of endogenous BRs in regulation of these processes (Figure 9). The analyzed, semi-dwarf NILs, which are defective in BR biosynthesis or signaling, displayed enhanced tolerance to drought. Moreover, the enhanced tolerance to water deficit exhibited by the semi-dwarf, BR mutants of barley is independent of the genetic background of the mutants. The obtained results indicate that perturbations in the BR metabolism negatively affect the profiles of glucose and fructose accumulation in a similar manner under both the control and stress conditions. Drought stimulates the accumulation of these monosaccharides, and the BR-defective barley mutants retain the capability to react to the stress accordingly. Under the control conditions, the genotype-dependent profile of sucrose accumulation was similar to the glucose and fructose accumulation profiles. However, in contrast to the profile of accumulation of these monosaccharides in reaction to drought, the stress conditions induced a significant reduction in the sucrose content in all the analyzed genotypes. It may be inferred that the significant increase in glucose and fructose accumulation in reaction to drought occurs at the expense of sucrose accumulation. 

Accumulations of the *HvAPX, HvCAT2* and *HvSOD1* transcripts encoding the antioxidant enzymes showed diverse profiles. The accumulation of the *HvAPX* and *HvSOD* transcripts under the control conditions is dependent on the BR biosynthesis and signaling. Under the stress conditions, accumulation of the *HvAPX* transcript is dependent on the BR metabolism, whereas accumulations of the *HvCAT* and *HvSOD* transcripts do not seem to be affected by the perturbations in the BR biosynthesis or signaling. Activities of the enzymatic antioxidants showed diverse profiles under both the control and drought conditions. The obtained results point to a significant role of BR biosynthesis and signaling in regulation of the HvAPX and HvCAT activities under the control conditions and the HvAPX and HvSOD activities during physiological reactions to the prolonged drought exposure. The results obtained in this study provide novel insights into the role of endogenous BRs in the regulation of metabolic reaction to drought as well as bring new questions which need to be answered. Particularly, it should be elucidated whether BR influences the stability of enzymatic antioxidants and which regulatory mechanisms may be involved. It would also be interesting to find out whether expression of genes encoding the enzymatic antioxidants can be regulated in a small-RNA-dependent manner. Another open question regards whether BR plays any role in epigenetic regulation of the antioxidant gene expression.

## Supplementary Materials

Supplementary Materials can be found at https://www.mdpi.com/1422-0067/21/14/5096/s1.
