# Supplementary material for: Insights into Metabolic Reactions of Semi-Dwarf, Barley Brassinosteroid Mutants to Drought
Source: Int J Mol Sci. 2020 Jul 19;21(14):5096. doi: 10.3390/ijms21145096 (PMC7404083; doi:10.3390/ijms21145096)
Supplement: Supplementary file 1 [file ijms-21-05096-s001.pdf]

Supplementary materials

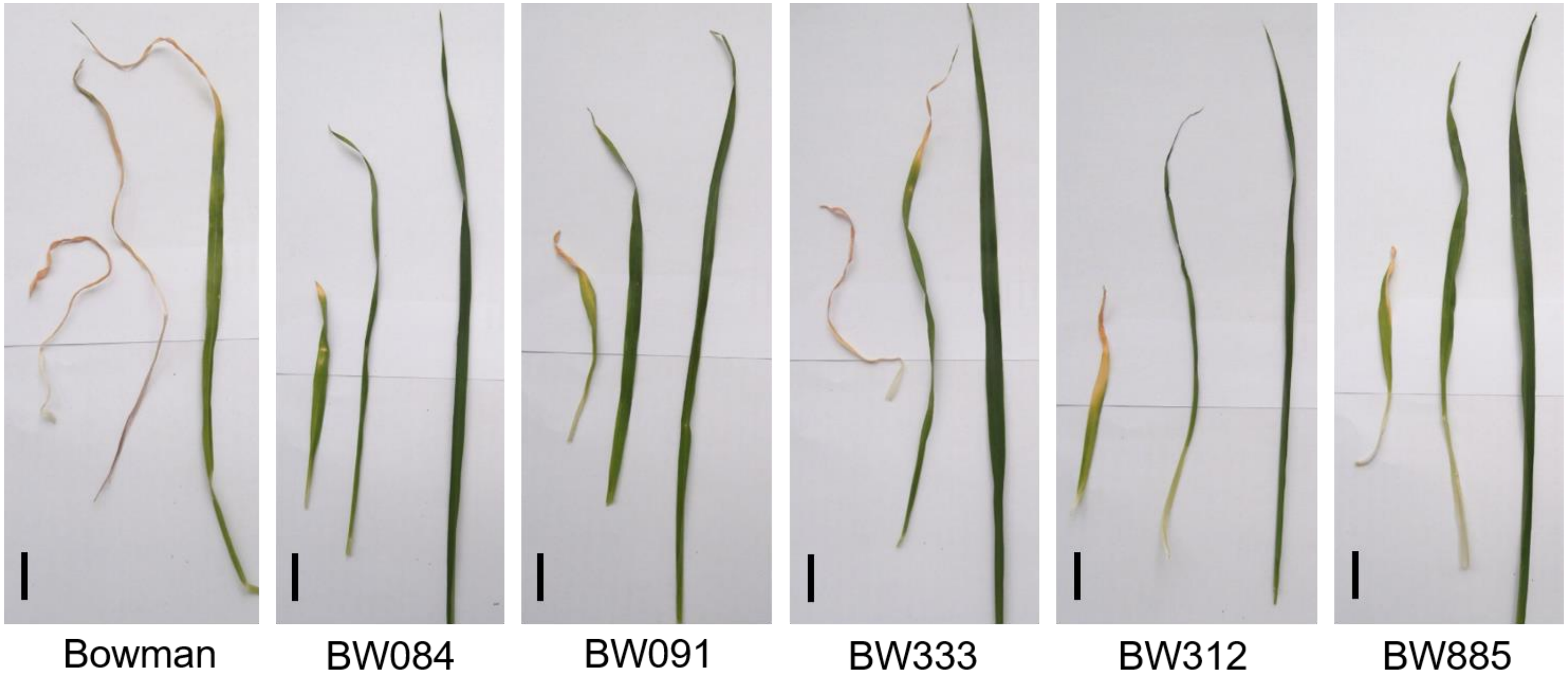

Figure 1S . Leaf injuries in the 'Bowman' and BR-deficient or BR-insensitive mutant plants after 3 weeks of drought. In each picture from left to right: the first (the oldest), the second, and the third leaf of each genotype. Bar: 1 cm.

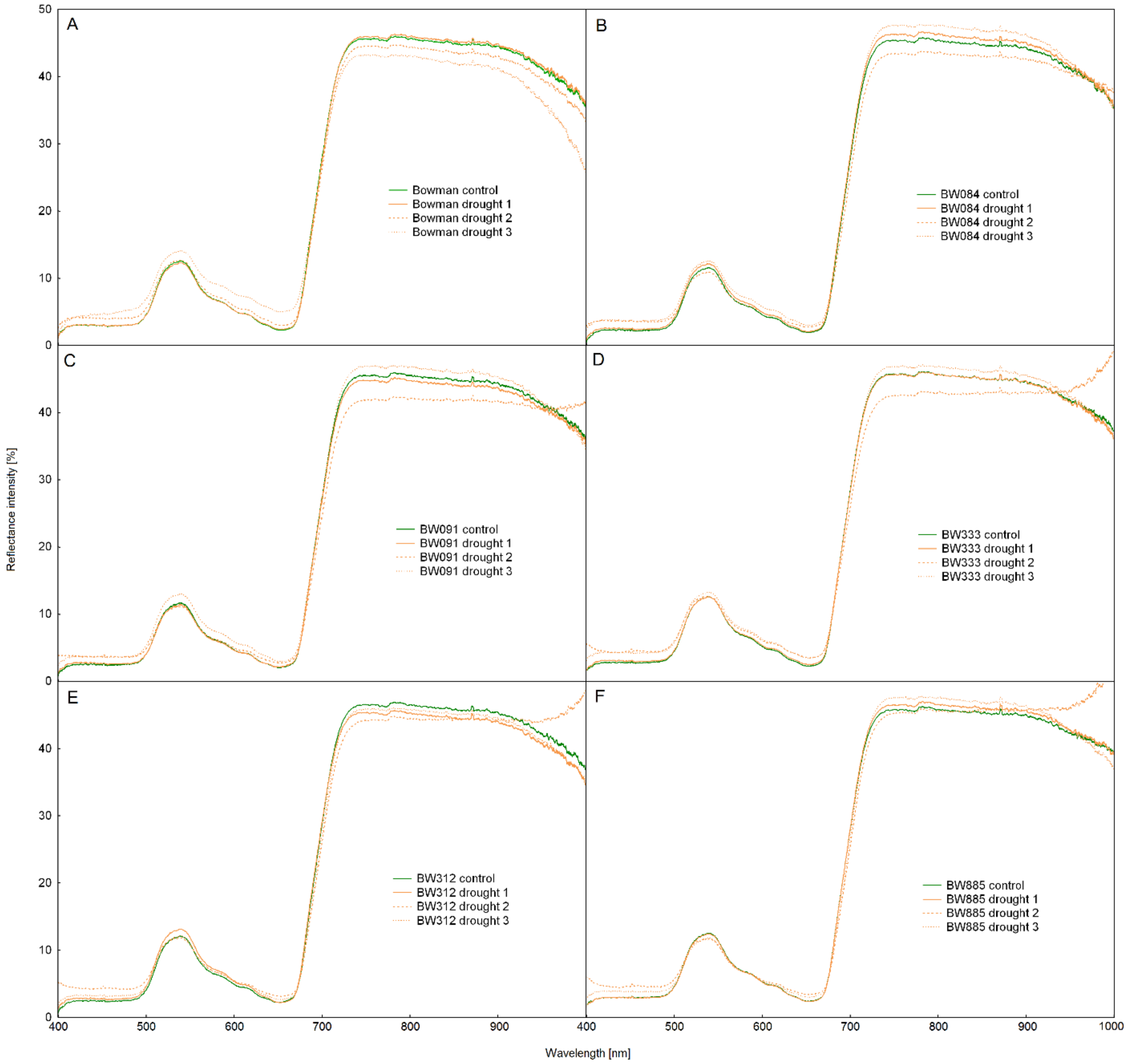

Figure 2S. The reflectance signatures of the analyzed genotypes under the control conditions and during the 3-week drought period.

Table 1S. Sequences of primers and probes used in the analysis of the *HvAPX*, *HvCat2*, and *HvSOD1* transcript accumulation. The *HvADP* gene was applied as the endogenous control.

**Gene name**	**GenBank ID**	**Forward primer**	**Reverse primer**	**TaqMan MGB Probe**
Cat2	U20778.1	CTTCAAGCCCAACCCAAAGTC	TGGTGGGAGAGGAAGTCGAA	FAM- ACGCGCCAGTACTC-MGB
SOD1	HM537232.1	GGTGACACGACTAATGGATGCATAT	CATGTGTCAGGCCGTTTGG	FAM- ACAGGGCCACATTTTA-MGB
APX	AJ006358.1	CGCCGATCTCTACCAGCTT	TCCCTCCCTGGGTGGAA	FAM- CTCCACGGCGACAACT-MGB
ADP	AJ508228.2	GTGGAGGCACTACTTCCAGAAT	ACAACACGTTCCCTATCATTGCT	FAM- ACGCAGGGCCTCATTT-MGB
